# The Role of *Saccharomyces cerevisiae* Yeast and Lactic Acid Bacteria in the Formation of 2-Propanol from Acetone during Fermentation of Rye Mashes Obtained Using Thermal-Pressure Method of Starch Liberation

**DOI:** 10.3390/molecules24030610

**Published:** 2019-02-09

**Authors:** Katarzyna Pielech-Przybylska, Maria Balcerek, Urszula Dziekońska-Kubczak, Barbara Pacholczyk-Sienicka, Grzegorz Ciepielowski, Łukasz Albrecht, Piotr Patelski

**Affiliations:** 1 Department of Spirit and Yeast Technology, Institute of Fermentation Technology and Microbiology, Faculty of Biotechnology and Food Sciences, Lodz University of Technology, Wolczanska 171/173, 90-924 Lodz, Poland; maria.balcerek@p.lodz.pl (M.B.); urszula.dziekonska-kubczak@p.lodz.pl (U.D.-K.); piotr.patelski@p.lodz.pl (P.P.); 2 Institute of Organic Chemistry, Faculty of Chemistry, Lodz University of Technology, Zeromskiego 116, 90-924 Lodz, Poland; barbara.pacholczyk@p.lodz.pl (B.P.-S.); grzegorz.ciepielowski@p.lodz.pl (G.C.); lukasz.albrecht@p.lodz.pl (L.A.)

**Keywords:** 2-propanol, acetone, thermal-pressure method of starch liberation, Maillard reaction products, *Saccharomyces cerevisiae* yeast, lactic acid bacteria

## Abstract

This study set out to assess the acetone content in rye sweet mashes prepared using the thermal-pressure method of starch liberation, and to investigate the formation of 2-propanol during the fermentation process. In the first set of experiments, we evaluated the correlation between the color and the content of acetone and furfural in industrially produced sweet mashes (*n* = 37). The L * value was negatively correlated with the content of both acetone and furfural, while chromatic parameters a * and b * and the yellowness index (YI) had strong positive correlations with acetone (r > 0.9) and furfural (r > 0.8 for a * and r > 0.9 for b * and YI). In the second set of experiments, we assessed the concentration of acetone and 2-propanol in distillery rye mashes, fermented by *S. cerevisiae* yeast and lactic acid bacteria. The influence of fermentation temperature on the formation of 2-propanol was also evaluated. The presence of 2-propanol in the post-fermentation media was confirmed, while a decrease in acetone content was observed. Fermentation temperature (27 °C or 35 °C) was found to have a significant effect on the concentration of 2-propanol in trials inoculated with lactic bacteria. The content of 2-propanol was more than 11 times higher in trials fermented at the higher temperature. In the case of yeast-fermented mashes, the temperature did not affect 2-propanol content. The acetone in the sweet mash was assumed to be a precursor of 2-propanol, which was found in the fermented mashes.

## 1. Introduction

Thermal-pressure processing is a common method in agricultural distilleries which use starchy raw materials. Its technological benefits include sterilization, which eliminates process-dangerous lactic acid bacteria on cereal grains [1], and the liberation of starch from plant cells. Moreover, high temperatures change the physical properties of starch, enabling more effective enzymatic hydrolysis. However, heat treatment of starchy materials can lead to a number of chemical changes, involving reducing sugars, amino acids, and peptides, which are abundant in cereal raw materials [2]. During heating, reactions may occur between the carbonyl or hemiacetal groups in reducing sugars and the amino groups in amino acids and peptides. These can then initiate a series of reactions, referred to collectively as Maillard reactions, which produce compounds with strong sensory properties. Maillard reaction products (MRPs) affect the taste, smell, and color of sweet mash obtained using the thermal-pressure method [3,4,5,6,7]. Moreover, Maillard reactions involve reducing sugars, which might otherwise have provided a potential substrate for ethanol production, as well as amino acids with peptides, which could have been a valuable nitrogen source for yeast in the fermentation process. Some MRPs, including furfural and 5-hydroxymethylfurfural (HMF), have been shown to affect the fermentation process [8,9,10,11]. Furfural and HMF are sugar degradation products, formed by dehydration of pentoses and hexoses, respectively, at high temperature and pressure [12,13]. Both are known to have a negative effect on *Saccharomyces cerevisiae* yeast. However, furfural is considered a much stronger inhibitor than HMF in terms of yeast growth and the productivity of ethanol. This is related to its effect on glycolytic activity and the tricarboxylic acid cycle, as well as to its causing oxidative stress and reducing the activity of various dehydrogenases in yeast cells [12,14]. Agricultural distilleries using starchy raw materials therefore need to control the steaming time carefully, to minimize the concentration of MRPs. 

Steaming of cereal raw materials—such as rye, wheat, or triticale—is carried out at pressures of 0.3–0.4 MPa and temperatures of 144–152 °C for about 30–40 min. The steaming of barley grain requires harsher parameters (0.5 MPa, 159 °C), due to the presence of an additional fibrous layer around the kernel, which develops as the flower buds grow together with the kernel during the grain’s puberty [15]. A well-steamed mass should have a light-yellow color, and the content of the kernel hulls should flow out freely.

Apart from furfural and 5-hydroxymethylfurfural (HMF), MRPs notably include aldehydes, for example phenylacetaldehyde and benzaldehyde, as well as pyrazines [16,17], other furans [18], pyridine, melanoidins [19], and ketones [6]. In research into the formation of carbonyl compounds in Maillard reactions, Rooney et al. [20] confirmed that, in the presence of amino acids—such as alanine, valine, leucine, isoleucine, phenylalanine, and methionine—the corresponding aldehydes are formed—i.e., acetaldehyde, isobutyraldehyde, isovaleraldehyde, 2-methylbutanal, phenylacetaldehyde, and methional. Moreover, small amounts of other carbonyl compounds may be detected, including acetone. Ketones were also identified among MRPs studied by Cui K. et al. [18]. Mansour et al. [21], in an investigation into the effects of glucose, fructose, and sucrose on the flavor of extruded wheat semolina, likewise detected ketones. 

In the next stage of the process, ethanol fermentation, the MRPs present in distillery mashes are transformed into other compounds by yeast. Aldehydes will be reduced to corresponding alcohols, which then participate in the synthesis of esters. Acetone is also reduced to the corresponding alcohol, in this case 2-propanol (syn. isopropyl alcohol). 2-propanol belongs to the group of higher alcohols. Higher alcohols are the dominant volatile compounds present in fermented mashes. Some of them, such as 2-methyl-1-butanol, 3-methyl-1-butanol, 2-methyl-1-propanol, 1-propanol, and 2-phenylethanol, are synthesized by the yeast from amino acids [22,23] and/or simple sugars [24,25]. However, the first pathway is dominant. Synthesis of other higher alcohols, such as 2-butanol, 2-propanol, 1-pentanol, and 1-hexanol, which occur in lower concentrations, only takes place via the latter pathway [26]. Bacteria are also able to synthesize secondary and primary higher alcohols [27,28]. Bacteria, as well as yeast, are present in distillery mashes [1,22]. The most common are lactic acid bacteria [29,30], for which close to optimal growth conditions occur during the fermentation process. Therefore, particular attention should be given to the temperature during fermentation and the initial pH of the sweet mash. High values for each of these parameters affect the growth of lactic acid bacteria [31], which poses a real threat to the proper course of the process, since acetic acid and lactic acid are well known yeast inhibitors [32,33]. Moreover, lactic acid bacteria are well known for their ability to synthesize volatile compounds such as alcohols, aldehydes, ketones, esters, alkanes, alkenes, terpenes, furans, and sulfur compounds [34,35,36,37]. Some of these negatively affect the quality of the agricultural distillates obtained [38,39]. 

In our previous research [40], we found 2-propanol in distillates obtained from rye- and potato-based mashes prepared using both thermal-pressure (TP) and pressureless (PLS) methods for starch liberation. It was noticed that the concentration of 2-propanol was substantially higher in distillates obtained from mashes prepared using the TP method. The low boiling point of 2-propanol compared to other higher alcohols (C3-C8) and its ability to form azeotropes makes the removal of this compound from the spirit during rectification very difficult. Therefore, it is necessary to limit its synthesis during fermentation. According to EU Regulations [41], the higher alcohols level (expressed in 2-methyl-1-propanol) in ethyl alcohol of agricultural origin is limited to a maximum of 0.5 g per hectoliter of 100% vol. alcohol, while to meet non-EU standards [42] the concentration of other alcohols, especially isopropyl alcohol, should be also taken into account, due to the difficulty of removing it in the distillation processes.

According to the literature [27,28,36,37], the synthesis of 2-propanol takes place via acetone reduction catalyzed by primary and/or secondary alcohol dehydrogenase. For acetone synthesis, in turn, the main substrate is acetyl-CoA. Two molecules of acetyl-CoA are condensed to one molecule of acetoacetyl-CoA (by acetoacetyl-CoA synthase), from which acetoacetate is formed (by acetoacetyl-CoA transferase) and finally acetoacetate is decarboxylated to acetone (by acetoacetate decarboxylase) [27]. Acetic acid, which is used by yeast and bacteria to synthesize acetyl-CoA, is already present in sweet mash, and is also formed during the fermentation process [1,43]. According to Martins et al. [5] and Davídek et al. [43], acetic acid is formed during Maillard reaction as the result of the degradation of Amadori products. Based on studies by Rooney et al. [20], Davidek et al. [43], and Cui et al. [18], it can further be assumed that acetone may be present in sweet mash (i.e., before fermentation) prepared by using the TP method, as a result of Maillard reactions. According to Davídek et al. [43], acetone formation occurs as a result of hydrolytic α-dicarbonyl cleavage of 2,4-pentanedione. However, the literature does not provide adequate reasons for the synthesis of 2-propanol during the ethanol fermentation of distillery sweet mashes. Differences between the technologies used for sweet mash production, as well as the complexity of the biochemical processes which occur during fermentations involving both yeast and other microorganisms (mainly lactic acid bacteria), combined with the action of multiple factors (including pH, temperature, sugars content, time), make it difficult to clearly explain the presence of 2-propanol. 

The purpose of the present study was therefore to evaluate whether and in what quantities acetone is present in distillery sweet mashes prepared using the TP method. We then investigated the effects of *S. cerevisiae* yeast and lactic acid bacteria on acetone metabolism in distillery mashes, and on 2-propanol content in the fermented mashes, depending on the fermentation temperature.

## 2. Results and Discussion

### 2.1. Colorimetric and Chromatographic Evaluation of Sweet Mashes

**Figure 1:**
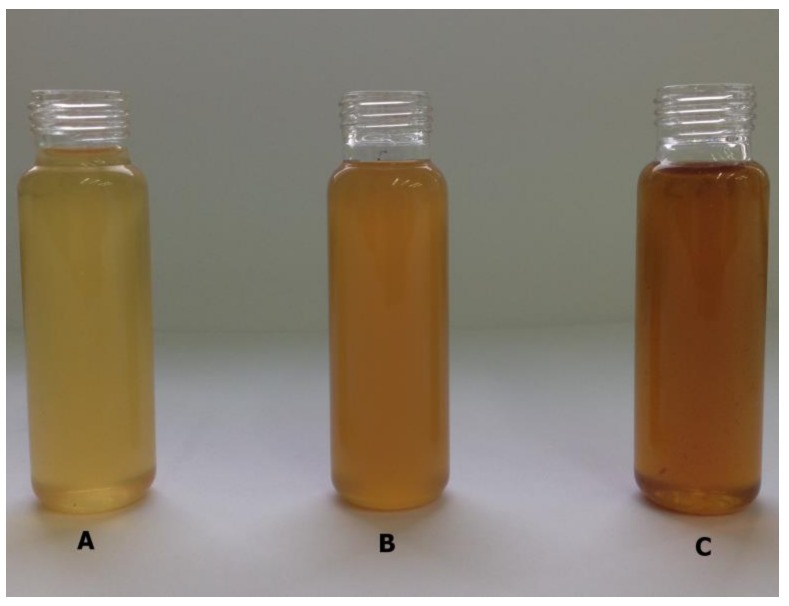
Color of sweet mash samples (**A**–sample no. 29; **B**–sample no. 22; **C**–sample no. 34).

In our previous study [40], we compared the concentrations of 2-propanol in distillates from mashes prepared using TP and PLS for starch liberation. We demonstrated that the method of processing the starchy raw materials had an effect on the level of isopropyl alcohol. In the present study, we attempted to identify acetone as a possible precursor for the synthesis of isopropanol in mashes prepared using the pressure-thermal method. Cui et al. [18] and Rooney et al. [20] had already reported the presence of acetone among MRPs. We evaluated 37 sweet mash samples prepared in an agricultural distillery. During the preparation of sweet mash samples for gas chromatographic analysis, we observed differences in the colors of some samples (Figure 1). 

We therefore undertook to assess the lightness and color of the sweet mashes. To confirm the connection between the observed differences in color and the composition of MRPs, the contents of furfural and HMF were also measured. Hydroxymetylfurfural and furfural are both indicators of Maillard reactions, which occur during the thermal processing of different raw materials. The former indicates reactions involving hexoses, while the latter indicates reactions involving pentoses [44]. In all 37 samples, only furfural was detected in the sweet mashes, and no HMF was observed. The concentrations of furfural in the mash samples were compared to the results of color and brightness measurements. Under the assumption that acetone, which has been identified as a product of Maillard reactions [18,20], is a potential precursor of 2-propanol and is formed under harsh TP conditions, its concentration and correlation with color was also evaluated. Acetone alone does not affect the color. However, it appears in parallel with colored MRPs as thermal treatment progresses. We therefore attempted to evaluate this correlation.

The color parameters and lightness measurements are shown in Table 1, while the results for furfural and acetone content are listed in Table 2. 

Analysis of variance showed statistically significant differences in the colors of the sweet mashes. The average values for L *, a *, and b * parameters ranged from 70.33 to 87.50, from −1.21 to 14.82, and from 78.30 to 42.60, respectively. Of the sweet mash samples, 27 showed positive a * parameter values, signifying a greater proportion of red pigment, as opposed to the remaining 10 samples, which had negative a * parameter values, indicating a higher content of green pigment. The differences in the positive values of this parameter ranged widely, from 0.51 (sample no. 4) to 14.82 (sample no. 34). The highest values for the a * parameter were observed in the cases of samples no. 7, 16, and 27. The values for parameter b * indicated a large share of yellow pigment in all the analyzed samples. However, as in the case of parameter a *, samples no. 7, 16, 27, and 34 had the highest values for this parameter, in the range of 71.33–78.30. Increases in the levels of red and yellow pigments were reflected in the brightness (L *) of the mash samples. The mashes with the highest values for color parameters a * and b * were the darkest. The brightness of samples 7, 16, 27, and 34 ranged from 70.33 to 74.34. The variations in the yellowness index (YI) of the analyzed mash samples were related to the differences in the values of the chromatic index b * and the brightness index L *. Statistically significant differences were found in the values of the calculated YI, which was also the highest for samples no. 7, 16, 27, and 34. 

Analysis of variance showed significant differences in the concentrations of acetone and furfural (Table 2). The content of these compounds was the highest in the samples with the darkest color parameters, in which the proportions of red and yellow were highest, i.e., samples no. 7, 16, 27, and 34. On this basis, it can be assumed that there is a relationship between the color of the sweet mash and the concentrations of acetone and furfural. 

Analysis of the correlations between parameters describing the brightness and color components of the sweet mash, the calculated yellowness index, and the acetone and furfural contents (Table 3) revealed a close relationship between these variables. Based on the calculated Pearson correlation coefficients (Table 3), the L * value (lightness or darkness) was negatively correlated with both the acetone and the furfural content. This confirms that increases in darkness (a lower L * parameter) are accompanied by higher concentrations of furfural and acetone. The strength of these dependencies was confirmed by a high correlation (r > 0.8).

The chromatic parameters a * and b * had strong positive correlations with acetone (r > 0.9) and furfural (r > 0.8 for a * and r > 0.9 for b *). This suggests that the concentrations of these compounds in the sweet mash rose with increases in the presence of red and yellow pigments. An equally strong positive correlation was observed in the case of the YI (r > 0.9). Arachchi et al. [45], Rooney et al. [20], and Echavarría et al. [46] evaluated the colors of model solutions subjected to heating and their contents of different MRPs. During the heating of glucosamine and cysteine mixtures, Arachchi et al. [45] observed that the Hunter’s b value increased rapidly at higher temperatures, which was associated with the content of MRPs. Rooney et al. [20], in their investigation into the formation of carbonyl compounds in model aqueous systems containing different sugars and amino acids, concluded that color intensity was highest in the presence of glucose with valine, leucine, isoleucine, lysine, phenylalanine, or methionine. In the trials with the highest color intensity, the same authors observed a higher concentration of carbonyl compounds, including acetone. A similar relationship was noted with xylose as a source of sugar. The most intense colors were related to the presence of valine, leucine, or lysine as an amino acid. Echavarría et al. [46] used a * and b * values to explain the color development of melanoidins. They noticed that the color development of the melanoidins was influenced by the type of amino acid and the temperature. Karnagwa et al. [16] reported the presence of ketones (including 2-butanone, 2-pentanone, and 2-heptanone) in model media containing free amino acids and peptides with xylose. Carbonyl compounds are well known products of the decomposition of lipid hydroperoxides, which are themselves products of lipid oxidation [47]. Both low and high molecular weight polymers affect nonzymatic browning [48].

The correlations (Table 3) and the regression model results (Table 4) suggest that higher levels of red and yellow pigments in sweet mash and higher values for YI are accompanied by increases in the concentrations of furfural and acetone. Based on the calculated R^2^ coefficients, it can be concluded that 80% of the variability in the concentrations of furfural and acetone can be associated with changes in the values of the color parameters for brightness and yellowness index. Regression equations can therefore be used to estimate the concentrations of acetone and furfural.

The significant differences observed in the colors of the sweet mashes from agricultural distillery are caused by a lack of repeatability during the evaporation process and by the high pressure associated with prolonged steaming. An important stage during the steaming of cereal raw materials is the periodic circulation of the content of the steamer. This is necessary to prevent the grain from overheating, because cereal grain has high bulk density. Circulation is achieved by lowering the pressure in the steamer. By opening the venting valve, the pressure is reduced slightly, which results in the cereal grains being mixed. The pressure and steaming time used in the agricultural distillery from which the sweet mash samples were collected (50 min at 0.42–0.45 MPa) was very long. It is likely that a shorter process time would reduce the concentration of MRPs. In their research, Kłosowski and Błajet-Kosicka [17] increased the pressure gradually during evaporation, to 0.5 MPa with 2–3 min of three-time circulation (at pressures of 0.15, 0.25, and 0.35 MPa). The maximum pressure of 0.5 MPa was maintained for only 5–10 min. A significant reduction in MRPs, in this case pyrazines, was observed compared to the control sample (0.5 MPa for 30 min, single circulation at 0.3 MPa).

### 2.2. Fermentation Results of Sweet Mashes Using Yeast and Lactic Acid Bacteria at Different Temperatures

In the second stage of the study, fermentations of rye distillery sweet mashes were carried out to assess the impact of temperature and the type of microorganisms on the content of acetone and 2-propanol in mashes after fermentation. The concentration of acetone in the laboratory-prepared sweet mash was 1.11 ± 0.04 mg/L (Table 5). The mash samples were inoculated using different microorganisms, *S. cerevisiae* yeast, or lactic acid bacteria strains, and then fermented in thermostatic rooms with different temperatures (air temperature 27 °C or 35 °C). The temperatures used in our experiments were selected based on observations (data not published) of the conditions used in agricultural distilleries. It was noticed that, as the temperature in the fermentation tanks increased (reaching up to 38–39 °C in the main phase of the fermentation process), the concentration of 2-propanol in the spirit also rose in comparison to samples of spirits obtained from mashes fermented at lower temperatures (maximum temperature in the fermentation tank 33–34 °C). Excessive temperatures during fermentation may be caused by setting a high temperature for the mash at the beginning of the process (over 30 °C), the lack of a cooling system for the fermentation tanks, or by ambient temperature, which is most often determined by weather conditions. 

The results of statistical analysis (Table 5) show that both the temperature and the microorganisms present in the mash affected the concentration of acetone and 2-propanol.

As can be seen, in comparison with the sweet mash (before fermentation) the samples fermented using *S. cerevisiae* yeast showed a decrease in the concentration of acetone. It is worth noting that the reduction was similar in both samples fermented by the yeast. Simultaneously, we detected 2-propanol in these samples, although the temperature did not affect its content. The presence of 2-propanol may confirm the reduction of acetone to isopropyl alcohol. Alcohol dehydrogenases (ADH, EC 1.1.1.1) react with a wide variety of alcohols, aldehydes, and ketones [49]. However, yeast alcohol dehydrogenases are the most active towards acetaldehyde and ethanol, while the oxidation of other alcohols (primary and secondary alcohols) and the reduction of higher aldehydes or methyl ketones catalyze poorly [50]. The lower activity of yeast ADH towards longer chain alcohols, aldehydes, and methyl ketones is due to the smaller size of the substrate binding pocket [51]. Probably for this reason, the acetone was not completely metabolized during the process of ethanol fermentation. Moreover, the temperature in the thermostatic rooms did not affect the ethanol content or fermentation efficiency (Table 5). 

Compared to yeast, bacteria are more often mentioned as producers of secondary alcohols, including isopropanol [52]. Clostridia is the most well researched species [36]. There is less information in the literature concerning lactic acid bacteria, although they are commonly found in distillery mashes [29]. The quantity of bacteria, including lactic acid bacteria, in mash depends on the method of starch liberation. However, there is always some quantity present [1,29]. Therefore, to ascertain whether lactic acid bacteria were responsible for the reduction of acetone to isopropanol, two mash samples were inoculated with two lactic acid bacteria strains, using antifungal protection (nystatin). A decrease in acetone concentration was observed, but it was highly dependent on the temperature. During fermentation at 27 °C, the concentration of acetone dropped by 0.22 mg/L of mash, while at 35 °C it decreased by 0.88 mg/L (by 79%). There were also differences in the concentration of 2-propanol in the mashes after 72 h, depending on the temperature in the thermostatic room. The content of 2-propanol was more than 11 times higher in mash fermented at the higher temperature. Lactic acid bacteria, depending on the genera and species, are categorized as either thermophiles or mesophiles. *Lactococcus lactis* ssp. *lactis* are included in the latter group. However, studies have shown [53] that increasing the temperature from 25 °C to 38 °C increases their specific growth rate. *Lactobacillus casei* bacteria belong to the group of thermophiles. The influence of temperature on the growth and metabolism of lactic acid bacteria was shown by the lactic acid content. The concentration of lactic acid in the mash inoculated with two strains of bacteria was 3.1 g/L when the temperature in the thermostat was set to 27 °C, and 5.2 g/L when the temperature in the thermostat was 35 °C (Table 5). 

In our previous research [1], we showed that during the fermentation of mashes not only yeast growth but also bacterial growth may be observed, including the growth of lactic acid bacteria. The source of the bacteria present during ethanol fermentation may be the starchy raw materials, but also the yeast inoculum. In agricultural distilleries, the yeast are propagated in a tank called a ‘yeast tank’, using sweet mash as a medium. To limit bacterial growth during yeast propagation, the initial pH of the sweet mash is set at 3.5–4.0. Propagation starts when the sweet mash is inoculated with dry distillery yeast (after rehydration). The cycle of yeast propagation takes about 10–12 h. Then about 80% of the volume of the inoculum in the yeast tank is used to inoculate the sweet mash in the fermentation tank. Next, a fresh portion of the sweet mash is added to the yeast tank and the next cycle of yeast propagation is carried out. An average of two cycles are carried out per day. How often the yeast is refreshed differs depending on the distillery. It may be every 2–4 weeks, or every 6 months. Lactic acid bacteria are categorized as neutrophils, although some strains show the ability to grow at lower pH. The composition of the medium can also have a significant effect on their survival at low pH values. LeBlanc et al. [54] observed a slowdown in the growth of *Lactobacillus fermentum* in medium at pH 4.5 compared to samples with a higher pH. Vrancken et al. [55] showed that the *Lactobacillus fermentum* IMDO 130201 strain converted sugars present in the medium (glucose, fructose, and sucrose) and form lactic acid in a pH range from 5 to 3.5. In our research, we also detected both lactic and acetic acids in mash samples collected from the yeast tank of an agricultural distillery after the 1st and 10th cycles of yeast propagation. The results (Table 6) show that lactic acid content was two-fold higher in the mash after the 10th cycle of propagation in comparison to the mash after the 1st cycle. A similar relationship was observed for acetic acid content. 

An increase in the concentrations of both acids may indicate the growth of lactic acid bacteria during yeast propagation. Inoculation of the sweet mash in the fermentation tank with yeast contaminated with bacteria may cause the growth not of only of yeast, but also of lactic acid bacteria. Moreover, distilleries usually initially set the sweet mash in the fermentation tank to pH 5 or over, which further promotes bacterial growth. 

## 3. Materials and Methods

### 3.1. Industrial Scale Pressure-Thermal Treatment and Mashing Process

Rye grains were treated on an industrial scale, in a tapered cylindrical steamer (total volume 3.75 m^3^). The steamer was filled with water (1.75 m^3^), which was heated to boiling point by injecting superheated steam. Then, 0.7 t of rye grains was poured in. After venting, the steamer was closed and the pressure inside was increased gradually to 0.42–0.45 MPa. The raw material was steamed at this pressure for 50 min, with periodical circulation of the content. The whole process was carried out with manual control of the steaming parameters (pressure and time) and circulation. Upon completion, the content of the steamer was transferred to a cylindrical steel-mashing vessel. 

The steamed mass was stirred continuously in a steel mashing vessel and cooled to 90 °C, at which point liquefying Termamyl S.C. preparation was added (0.13 L/t of starch, Novozymes, Bagsværd, Denmark). The mixture was kept at this temperature for 15 min, then cooled to 65 °C before the addition of saccharifying SAN Extra preparation (0.6 L/t of starch, Novozymes, Denmark). After the addition of SAN Extra, the mash was cooled further, to 32–34 °C. This procedure was repeated 37 times. All the mash samples (*n* = 37) were frozen immediately for transportation from the agricultural distillery to the laboratory. The mash samples were assessed in terms of their color and chemical composition. 

Mash samples from the yeast tank used for yeast propagation were also frozen and transported to the laboratory (after the 1st and 10th cycles). Prior to inoculation with the yeast, the sweet mash was acidified to pH 3.5. 

### 3.2. Laboratory Scale Pressure-Thermal Treatment, Mashing, and Fermentation Processes

Pressure-thermal processing and mashing (with enzyme preparations) were performed according procedures described previously [1]. Rye grains were used as the raw material. The samples of mash produced on a laboratory scale were used immediately to prepare fermentation trials. 

The pH of the sweet mash before fermentation was set at 5.0 using sulfuric acid (25% *w*/*w* solution). Two variants of the sweet mash were then prepared, with different microorganisms used for inoculation:

- Ethanol Red yeast (*Saccharomyces cerevisiae*, Fermentis Division S.I. Lesaffre, Marcq-en-Barœul, France) (1.3×10^7^ CFU/mL of mash), with the addition of 80 mg/L IsoStab^®^ hop α-acid preparation (BetaTec GmbH, Nürnberg, Germany), to protect the process from bacterial contamination and prevent microbial infections;
- a mixture of lactic acid bacteria strains *Lactococcus lactis* ssp. *lactis* ŁOCK0877 and *Lactobacillus casei* ŁOCK0901, obtained from the ŁOCK Pure Culture Collection (final quantity 4.0×10^6^ CFU/mL of mash), with the addition of nystatin, to prevent yeast growth.

Before fermentation, all of the mash samples (1000 mL) were supplemented with diammonium phosphate (0.2 g/L). The glass flasks (2000 mL) were closed with a fermentation tube filled with glycerin. Fermentations were performed in two thermostatic rooms, with the temperatures set to 27 and 35 °C. Fermentation was continued for 72 h. Before and after fermentation, the mash samples were taken for gas chromatographic analysis (HS-GC-MS). All experiments were prepared in triplicate.

### 3.3. Color Measurement

Tests were performed using a CHROMA METER CR-5 device (Konica Minolta, Osaka, Japan), using the CIE Lab scale. Three color components were measured: L* (darkness or lightness of color ranges from black (0) to white (100)), a* (+red to −green chromatic components), and b* (+yellow to −blue chromatic components). Based on the L* and b* parameters, the yellowness index (YI) was calculated as Equation (1) [56]


YI = 142.86b*/L*
(1)

Prior to analysis, samples of the mashes were centrifuged at 23,300× *g* for 10 min at 15 °C. The final result was the arithmetic mean of three measurements for each sample.

### 3.4. Determination of Total Sugars and Extract Content in the Mashes

The concentration of total sugars in the sweet and fermented mashes prepared on the laboratory scale was determined according to the method described previously [1]. The content of extract in the sweet mashes prepared at both the industrial and laboratory scales was measured using a hydrometer, according to the method described previously [57].

### 3.5. HPLC Analysis of Fermented Mashes

The concentrations of ethanol and lactic acid in the mashes were evaluated using HPLC, according procedures described previously [1].

Fermentation efficiency was calculated according to method described previously [57].

### 3.6. Gas Chromatographic Analysis (HS-GC-MS) of Sweet and Fermented Mashes

Qualitative analysis was performed using a GC apparatus (Agilent 7890A, Agilent Technologies, Santa Clara, CA, USA) coupled to a mass spectrometer (Agilent MSD 5975C, Agilent Technologies, Santa Clara, CA, USA). A capillary column was used to separate the compounds (TGWAX-MS, Thermo, Scientific Fisher, Pittsburgh, PA, USA; 60 m × 0.32 mm × 0.50 μm). The GC oven temperature was programmed to increase from 40 °C (5 min) to 80 °C at a rate of 5 °C/min, then to 220 °C at a rate of 10 °C/min, where it was maintained for 5 min. The flow rate of the carrier gas (helium) through the column was 1.1 mL/min. The temperature of the injector (split/splitless) was 250 °C. Injections of the tested samples were made in the split mode (10:1) using a headspace analyzer (Agilent 7697A, Agilent Technologies, Santa Clara, CA, USA). The temperatures of the MS ion source, transfer line, and quadrupole were 230, 250, and 150 °C, respectively. The ionization energy was 70 eV. Prior to analysis, a 20 mL headspace vial was filled with 7 mL of mash and closed tightly using an aluminum cap and septa. 

Headspace conditions:

- Temperature settings: oven temperature 50 °C, loop temperature 60 °C, transfer line temperature 70 °C.
- Timing settings: vial equilibration time 20 min, injection duration 0.7 min, GC cycle time 47 min.
- Vial and loop settings: vial shaking 136 shakes/min, fill pressure 15 psi, vial pressurization gas helium.

Acetone, MRPs (furfural and hydroxymethylfurfural), and 2-propanol were identified based on a comparison of their mass spectra with those of standard compounds and with the mass spectra in the NIST/EPA/NIH Mass Spectra Library (2012; Version 2.0g). 

Quantitative analysis of the sweet and fermented mashes was performed to determine the concentrations of the identified compounds. Headspace and GC conditions were the same those used for qualitative analyses. The acetone, furfural, and 2-propanol were quantified using calibration curves in the selected ion monitoring mode (SIM). Quantitative analysis was performed using Agilent MassHunter software (Agilent Technologies, Santa Clara, CA, USA). The results were expressed in mg/L of mash. All analyses were performed in triplicate.

### 3.7. Statistical Analysis

Statistical calculations were performed using STATISTICA 6.0 software (Tibco Software, Palo Alto, CA, USA). To evaluate the differences between the tested sweet mash samples, analysis of variance (ANOVA) was conducted with a 0.05 significance level. When statistical differences were detected (*p* < 0.05), means were compared using the post hoc Duncan test with a 0.05 significance level. Correlation and regression analyses were used to determine the relationship between the test color components and the concentrations of acetone and furfural. Significance tests were performed with a 0.05 significance level.

## 4. Conclusions

The purpose of this study was to evaluate the acetone content in distillery rye mashes prepared using the TP method. Mash samples prepared using the same method (from the same agricultural distillery) were found to differ significantly in color. This color variance was correlated to the concentration of acetone, which may be explained in part by the presence of furfural, an indicator of Maillard reactions. The concentrations of acetone and furfural increased with higher proportions of red (a *) and yellow (b *) pigments. Next, we investigated the content of 2-propanol in the mashes, after fermentation with *Saccharomyces cerevisiae* yeast and lactic acid bacteria. Based on our results, it is concluded that one of the reasons for the presence of 2-propanol in fermented mash may be the acetone in sweet mash prepared using the thermal-pressure method for starch liberation. When the acetone present in the sweet mash was reduced during fermentation, 2-propanol was observed in the fermented mashes. However, the presence of lactic acid bacteria can affect the increase in the concentration of 2-propanol, especially when the temperature during fermentation rises to the optimal values for their growth and activity.

## Figures and Tables

**Table 1:** Color parameters of the sweet mash samples^*^

Sample number	L*		a*		b*		YI	
	Mean	SD	Mean	SD	Mean	SD	Mean	SD
1	83.15_ghijkl_	2.58	2.08_j_	0.06	52.23_hij_	1.62	89.93_hi_	2.79
2	85.88_ijklm_	2.05	-0.53_d_	0.01	40.20_abc_	0.96	66.32_abc_	1.58
3	84.48_ghijklm_	2.27	1.37_hi_	0.04	51.68_ghi_	1.39	87.72_gh_	2.35
4	82.70_efghij_	1.91	2.53_k_	0.06	54.24_jkl_	1.25	93.89_ij_	2.17
5	84.47_ghijklm_	2.01	0.51_f_	0.01	45.12_e_	1.08	75.67_ef_	1.80
6	85.20_hijklm_	2.28	0.52_f_	0.01	46.23_e_	1.23	77.69_f_	2.08
7	72.01_ab_	1.66	14.06_t_	0.32	78.30_u_	1.81	155.66_r_	3.59
8	87.59_m_	2.34	-1.21_ab_	0.03	42.60_cd_	1.14	69.64_cd_	1.86
9	81.50_efgh_	1.63	3.93_m_	0.08	57.89_n_	1.16	101.47_k_	2.03
10	82.79_fghij_	2.28	2.15_j_	0.06	52.05_hij_	1.43	89.96_hi_	2.47
11	84.26_ghijklm_	2.61	0.78_g_	0.02	49.30_fg_	1.53	83.75_g_	2.60
12	83.66_ghijklm_	1.59	1.14_hi_	0.02	49.17_f_	0.94	83.90_g_	1.60
13	82.69_efghij_	1.97	1.55_i_	0.04	49.83_fgh_	1.19	85.38_g_	2.03
14	81.34_efgh_	1.88	3.31_l_	0.08	55.26_klm_	1.27	96.92_jk_	2.23
15	82.39_efghi_	1.77	2.62_k_	0.06	53.60_ijk_	1.15	93.10_ij_	2.00
16	70.90_ab_	2.13	14.15_t_	0.43	74.93_t_	2.26	150.68_p_	4.54
17	78.76_def_	2.21	6.03_n_	0.17	61.73_o_	1.73	112.40_l_	3.16
18	81.61_efgh_	2.25	3.74_m_	0.10	57.48_mn_	1.58	100.86_k_	2.77
19	78.71_de_	2.17	5.95_n_	0.16	62.70_o_	1.72	114.06_l_	3.14
20	76.10_cd_	2.04	9.10_r_	0.24	68.88_r_	1.85	129.79_n_	3.48
21	81.73_efgh_	2.13	3.27_l_	0.09	56.08_lmn_	1.46	98.33_k_	2.56
22	77.10_cd_	1.71	7.83_o_	0.17	65.81_p_	1.46	121.96_m_	2.71
23	81.54_efgh_	1.88	3.43_l_	0.08	56.81_mn_	1.31	99.73_k_	2.30
24	80.95_efg_	1.77	3.82_m_	0.08	57.02_mn_	1.25	100.60_k_	2.20
25	86.73_jklm_	2.37	-0.76_de_	0.02	41.59_bc_	1.14	68.81_bcd_	1.88
26	83.02_ghijk_	2.22	0.75_g_	0.02	41.90_bc_	1.12	72.26_de_	1.93
27	74.37_bc_	1.99	11.02_s_	0.29	71.33_s_	1.91	137.31_o_	3.67
28	87.55_m_	2.10	-1.24_ab_	0.03	38.32_a_	0.92	62.43_a_	1.50
29	87.01_klm_	2.40	-1.10_b_	0.03	40.61_abc_	1.12	66.86_abc_	1.84
30	87.18_lm_	2.44	-1.25_ab_	0.03	39.60_ab_	1.11	65.00_ab_	1.82
31	84.09_ghijklm_	1.87	-0.16_e_	0.00	44.56_de_	0.99	75.65_ef_	1.68
32	86.69_jklm_	1.99	-0.85_c_	0.02	39.56_ab_	0.91	65.26_abc_	1.50
33	87.58_m_	2.27	-1.43_ab_	0.04	38.14_a_	0.99	62.44_a_	1.62
34	70.33_a_	1.39	14.82_u_	0.29	76.95_tu_	1.52	156.02_r_	3.09
35	83.75_ghijklm_	2.24	1.58_i_	0.04	50.55_fgh_	1.35	86.42_gh_	2.31
36	76.56_cd_	2.04	8.47_p_	0.23	66.02_p_	1.76	123.48_m_	3.30
37	86.79_jklm_	2.32	-0.71_cd_	0.02	40.48_abc_	1.08	66.78_abc_	1.78

a–t—Mean values in the column with different lower-case letters are significantly different (ANOVA at significance level < 0.05); * Average extract content (i.e., soluble solids content) 17.2 ± 0.5 °Blg.

**Table 2:** Furfural and acetone content in sweet mash samples.

Sample number	Furfural (mg/L)		Acetone (mg/L)	
	Mean	SD	Mean	SD
1	3.07_g_	0.10	1.35_l_	0.04
2	2.01_de_	0.05	0.78_cde_	0.02
3	4.09_i_	0.11	1.06_h_	0.03
4	4.30_ij_	0.10	1.07_h_	0.02
5	2.19_ef_	0.05	0.81_de_	0.02
6	0.32_a_	0.01	1.03_gh_	0.03
7	22.73_t_	0.52	1.91_p_	0.04
8	4.00_i_	0.11	1.08_h_	0.03
9	6.47_l_	0.18	1.24_jk_	0.03
10	4.58_j_	0.13	1.18_ij_	0.03
11	4.03_i_	0.11	1.24_ijk_	0.03
12	2.87_g_	0.08	1.27_k_	0.04
13	3.67_h_	0.10	1.18_i_	0.03
14	2.29_ef_	0.06	1.22_ijk_	0.03
15	2.51_f_	0.07	0.99_g_	0.03
16	18.80_s_	0.52	1.91_p_	0.05
17	7.68_m_	0.21	1.18_ij_	0.03
18	6.15_k_	0.17	1.02_gh_	0.03
19	7.46_m_	0.21	1.46_m_	0.04
20	10.23_n_	0.28	1.66_n_	0.05
21	4.30_ij_	0.12	1.46_m_	0.04
22	6.67_l_	0.18	1.84_o_	0.05
23	3.20_g_	0.09	1.22_ijk_	0.03
24	2.50_f_	0.07	1.02_gh_	0.03
25	1.85_d_	0.05	0.74_bc_	0.02
26	0.45_ab_	0.01	0.76_cd_	0.02
27	11.67_p_	0.32	2.20_r_	0.06
28	2.19_ef_	0.06	0.83_e_	0.02
29	2.35_ef_	0.06	0.69_b_	0.02
30	0.58_ab_	0.02	0.77_cd_	0.02
31	1.18_c_	0.03	0.91_f_	0.03
32	0.69_b_	0.02	0.59_a_	0.02
33	0.47_ab_	0.01	0.61_a_	0.02
34	15.49_r_	0.43	2.40_s_	0.07
35	3.17_g_	0.09	1.05_h_	0.03
36	11.11_o_	0.31	1.23_ijk_	0.03
37	3.02_g_	0.08	0.75_bc_	0.02

a–t—Mean values with different lower-case letters are significantly different (ANOVA at significance level < 0.05).

**Table 3:** Pearson correlation coefficients (r) between color parameters and furfural and acetone content in sweet mashes.

Compound	L*		a*		b*		YI	
	*R*	*p*-Value	*r*	*p*-Value	*r*	*p*-Value	*r*	*p*-Value
Furfural	−0.8955	0.0000	0.9297	0.0000	0.8832	0.0000	0.9105	0.0000
Acetone	−0.8963	0.0000	0.9018	0.0000	0.9043	0.0000	0.9117	0.0000

**Table 4:** Linear regression results

Pairs of Relationships	*r*	R^2^	F	*p*-Value	Regression Equation
Furfural vs. L*	−0.8955	0.8020	141.7559	0.0000	Furfural = 84.9769 − 0.9738 * L*
Furfural vs. a*	0.9297	0.8644	223.0314	0.0000	Furfural = 1.7217 + 1.0442 * a*
Furfural vs. b*	0.8832	0.7800	124.0878	0.0000	Furfural = -15.8436 + 0.3944 * b*
Furfural vs. YI	0.9105	0.8291	169.7743	0.0000	Furfural = −11.3539 + 0.175 * YI
Acetone vs. L*	−0.8963	0.8034	143.0305	0.0000	Acetone = 7.9397 − 0.0824 * L*
Acetone vs. a*	0.9018	0.8132	152.3914	0.0000	Acetone = 0.9003 + 0.0857 * a*
Acetone vs. b*	0.9043	0.8178	157.0876	0.0000	Acetone = −0.6367 + 0.0342 * b*
Acetone vs. YI	0.9117	0.8311	172.2824	0.0000	Acetone = −0.2161 + 0.0148 * YI

**Table 5:** Acetone, 2-propanol, ethanol, and lactic acid contents in the sweet and fermented mashes

Sample	Temperature (°C)	Microorganism Added to Sweet Mash (Before Fermentation)	Compound Name	Concentration	
				Mean (*n*=3)	SD
Sweet mash^*^	-	-	Acetone (mg/L)	1.11_c_	0.04
			2-Propanol (mg/L)	nd	-
Fermented mash^**^	27	Yeast *S. cerevisiae* (with addition of α-hop acids)	Acetone (mg/L)	0.86_b_	0.04
			2-Propanol (mg/L)	0.23_b_	0.01
			Ethanol (g/L)	54.5_B_	4.2
			Lactic acid (g/L)	nd	-
		Lactic acid bacteria (with addition of nystatin)	Acetone (mg/L)	0.89_b_	0.04
			2-Propanol (mg/L)	0.07_a_	0.01
			Ethanol (g/L)	0.8_A_	0.0
			Lactic acid (g/L)	3.1_A_	0.4
Fermented mash^***^	35	Yeast *S. cerevisiae* (with addition of α-hop acids)	Acetone (mg/L)	0.80_b_	0.04
			2-Propanol (mg/L)	0.27_b_	0.01
			Ethanol (g/L)	52.9_B_	4.4
			Lactic acid (g/L)	nd	-
		Lactic acid bacteria (with addition of nystatin)	Acetone (mg/L)	0.23_a_	0.02
			2-Propanol (mg/L)	0.79_c_	0.04
			Ethanol (g/L)	1.2_A_	0.1
			Lactic acid (g/L)	5.2_B_	0.6

nd—Not detected; SD—Standard deviation. ^*^ Extract of sweet mash (i.e., soluble solids content) and total sugars content 14.3 ± 1.1 °Blg and 114.0 ± 6.8 g of glucose/L, respectively. ^**^ Total sugars content 4.15 ± 0.3 g of glucose/L (yeast) and 47.35 ± 3.1 g of glucose/L (lactic acid bacteria); Fermentation efficiency for yeast-provided fermentation reached 88 ± 4 % of the theoretical yield. ^***^ Total sugars content 1.30 ± 0.1 g of glucose/L (yeast) and 19.46 ± 2.4 g of glucose/L (lactic acid bacteria); Fermentation efficiency for yeast-provided fermentation reached 87 ± 3 % of the theoretical yield. a–c—Mean values for acetone with different letters are significantly different (results of two-way ANOVA with post-hoc Duncan’s multiple test, p<0.05). **a**–**c**—Mean values for 2-propanol with different letters are significantly different (results of two-way ANOVA with post-hoc Duncan’s multiple test, *p* < 0.05). A–B—Mean values for ethanol with different letters are significantly different (results of two-way ANOVA with post-hoc Duncan’s multiple test, *p* < 0.05). **A**–**B**—Mean values for lactic acid with different letters are significantly different (results of one-way ANOVA, *p* < 0.05). 
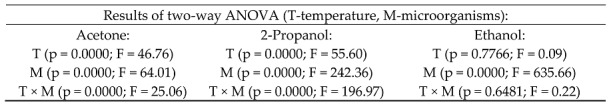

**Table 6:** Lactic acid and acetic acid content in mashes after the first and the tenth cycles of yeast inoculation (industrial scale).

Cyle of Yeast Inoculation	Compound Name	Concentration	
		Mean (*n* = 3)	SD
1st	Lactic acid (g/L)	1.1_a_	0.2
	Acetic acid (g/L)	0.4_A_	0.1
10th	Lactic acid (g/L)	2.1_b_	0.2
	Acetic acid (g/L)	0.7_B_	0.1

SD—Standard deviation. a-b—Mean values of lactic acid in column with different lower-case letters are significantly different (results of one-way ANOVA, *p* < 0.05); A-B—Mean values of acetic acid in column with different upper-case letters are significantly different (results of one-way ANOVA, *p* < 0.05).
